# Planetary Gearboxes Fault Diagnosis Based on Markov Transition Fields and SE-ResNet

**DOI:** 10.3390/s24237540

**Published:** 2024-11-26

**Authors:** Yanyan Liu, Tongxin Gao, Wenxu Wu, Yongquan Sun

**Affiliations:** 1 School of Mechanical Engineering, University of Science and Technology Beijing, Beijing 100083, China; gao18810580963@gmail.com; 2 Institute of Sensor and Reliability Engineering, Harbin University of Science and Technology, Harbin 150080, China; 2220610100@stu.hrbust.edu.cn

**Keywords:** Markov transfer fields, residual networks, gearbox fault diagnosis, attention mechanisms

## Abstract

The working conditions of planetary gearboxes are complex, and their structural couplings are strong, leading to low reliability. Traditional deep neural networks often struggle with feature learning in noisy environments, and their reliance on one-dimensional signals as input fails to capture the interrelationships between data points. To address these challenges, we proposed a fault diagnosis method for planetary gearboxes that integrates Markov transition fields (MTFs) and a residual attention mechanism. The MTF was employed to encode one-dimensional signals into feature maps, which were then fed into a residual networks (ResNet) architecture. To enhance the network’s ability to focus on important features, we embedded the squeeze-and-excitation (SE) channel attention mechanism into the ResNet34 network, creating a SE-ResNet model. This model was trained to effectively extract and classify features. The developed method was validated using a specific dataset and achieved an accuracy of about 98.1%. The results demonstrate the effectiveness and reliability of the developed method in diagnosing faults in planetary gearboxes under strong noise conditions.

## 1. Introduction

The planetary gear transmission system consists of the sun gear, planetary gears, planetary carrier, and ring gear [1], and has a series of advantages such as lightweight, small size, large transmission ratio, strong bearing capacity, and high transmission efficiency [2]. These benefits make it widely used in mechanical transmission systems in fields such as engineering machinery, wind power generation, aviation, and vehicles. Its operating conditions are harsh, complex, and variable, and the structural coupling is strong, making it prone to failure. Once a failure or performance degradation occurs, it can easily lead to a shutdown of the entire mechanical system [3]. The significance of fault diagnosis in planetary gearboxes cannot be overstated. Early detection of faults through advanced diagnostic techniques allows for the prevention of catastrophic failures, thereby mitigating the risk of equipment damage, production downtime, and potential safety hazards. By enabling proactive maintenance, fault diagnosis significantly reduces maintenance costs and minimizes the likelihood of unexpected breakdowns. This proactive approach not only extends the lifespan of the gearbox but also ensures that it operates at peak efficiency, maintaining the desired transmission performance. Furthermore, continuous monitoring and timely diagnosis provide valuable data-driven insights, helping to identify trends and potential issues before they escalate. This not only enhances the gearbox’s reliability but also ensures compliance with industry standards. However, frequent operation under low-speed, heavy-load conditions subjects the gear tooth surfaces to alternating loads, causing key components to suffer from frequent pitting, cracks, and other failures. Therefore, selecting efficient signal processing methods and accurately extracting fault characteristic information from the planetary gearbox is crucial for effective fault diagnosis.

Similar issues are encountered in fault identification for some periodic structures. When a rotating machine experiences a partial failure, the vibration signal contains periodic fault transients resulting from mechanical collisions among the rotating parts [4]. The gear transmission system in high-speed trains is responsible for delivering the power from the motor to the wheels, and is prone to periodic failures due to the wear and fatigue during the meshing of gear pairs and the transmission of torque [5]. Periodic fault transients serve as a prominent indicator of the status of a rotating machinery, so the extraction of these transients is a crucial step in the process of fault detection and condition monitoring.

Vibration signal analysis encompasses time-domain analysis, frequency-domain analysis, and time–frequency domain analysis, and forms the foundational basis for the characteristic analysis of rotating machinery [6]. Wang et al. proposed diversity entropy to estimate the complexity of the impulse data by combining the angular distance and relative probability distributions of similarities [7]. Li et al. introduced symbolic dynamic filtering into the entropy method, which has merits in robust noise influence and calculation efficiency [8]. Vibration analysis necessitates extensive knowledge of signal processing, and manual feature extraction involves a heavy workload, strong subjectivity, and fault identification that relies heavily on human experience, often affecting the generalization ability of the model [9]. To circumvent the limitations of vibration analysis, data-driven methods have gradually developed and been applied. With the advent of the big data era and the continuous advancement of deep learning, many scholars have applied deep learning to the fault diagnosis of planetary gearboxes. Pang et al. [10] utilized convolutional neural networks (CNNs) and vibration bispectra for fault diagnosis of planetary gearboxes, addressing the issue of low accuracy under multiple operating conditions. Li et al. developed a fault-sensitive region detection method based on infrared thermal imaging and deep learning algorithms to extract fault characters under variable conditions [11] Wang et al. combined CNNs with recurrence plots for fault diagnosis of planetary gearbox bearings [12]. However, one-dimensional CNNs have limitations in detecting local correlations in signals, and using one-dimensional signals as inputs to neural networks makes it difficult to fully utilize data correlations. Scholtyssek et al. employed balanced accuracy to train and evaluate convolutional neural networks [13], while Chen et al. integrated convolutional neural networks with discrete wavelet transform for fault diagnosis of planetary gearboxes, which can provide a more prominent and comprehensive time–frequency distribution representation [14]. CNNs have demonstrated promising results in the field of fault diagnosis for rotating machinery.

Although CNNs have been improved in terms of precision and universal applicability capabilities compared with manual feature extraction or traditional machine learning methods, they still face challenges in distinguishing features under complex operating conditions and strong noise scenarios [15]. To enhance the capability for learning features from high-noise vibration signals., Wang et al. applied minimum entropy deconvolution (MED) to the fault diagnosis of gearboxes, using simulated periodic transient signals with different signal-to-noise ratios (SNRs), and the excellent performance of MED in noise suppression was evaluated, revealing the shortcomings of extracting multiple pulses [16]. Zhao et al. applied residual networks (ResNet) to the fault diagnosis of gearboxes, and addressed the issue of traditional neural networks learning noise features as the network depth increases [17]. Zhang proposed a method to diagnose the fault of the planetary gearbox under the background of strong noise based on the adaptive loss-weighted element residual network [18]. This significantly improved the feature learning capability for strong noise vibration signals. The SE channel attention mechanism has been widely applied in tasks such as image classification, object detection, and image segmentation, achieving significant performance gains [19]. Wu et al. experimentally proved that the classification accuracy of two-dimensional convolutional neural networks is better than that of one-dimensional convolutional neural networks [20]. It enables the network to focus on the most salient features, making it more robust and effective in various applications.

It is challengeable to effectively separate and extract fault signals, making it difficult to accurately identify periodic faults. Furthermore, the correlation between various model feature channels has not been adequately considered, leading to inaccuracies in the network’s identification of noise features and fault features, and subsequently causing errors. To address these issues, this paper proposes a planetary gearbox fault diagnosis method that combines the channel attention mechanism with Markov transition fields. Firstly, one-dimensional vibration signals are converted into two-dimensional image signals that are easier to recognize through Markov image encoding. Then, the SE attention mechanism network (SEnet) is embedded into the ResNet network to form the SE-ResNet network. This network can adaptively learn the importance weights of each channel to adjust the activation values of each channel in the input feature map. At the same time, the advantages of the ResNet network itself are preserved, as each layer has a direct mapping component that preserves the original characteristics of the signal, enabling effective identification of periodic fault pulses. This solution addresses the difficulty of identifying periodic fault features in the presence of strong noise. In the experimental analysis, MTF-SE-ResNet is compared with CNN, ResNet34, and MTF-CNN, demonstrating the advantages of MTF-SE-ResNet in terms of diagnostic accuracy, iteration convergence speed, and anti-interference capability.

## 2. Engineering Background and Vibration Data Processing

### 2.1. Engineering Background and Problems

Planetary gearboxes differ from fixed-axis transmission gearboxes, where each gear rotates around its fixed central axis. A planetary gear transmission system comprises a sun gear, multiple planet gears, an internal gear ring, and a planet carrier. Usually, the internal gear ring stays fixed, as the sun gear spins around its own center. Meanwhile, the planet gears not only rotate around their central axes but also revolve around the central axis of the sun gear, engaging in conjunction with both the sun gear and the internal gear ring. Consequently, the movement of the gears in a planetary gearbox is distinguished by its unique combined motion, leading to a more complex vibration response compared with fixed-axis transmission gearboxes. As a result, the corresponding fault diagnosis issues possess unique characteristics and difficulties. The unique rotational characteristics of planetary gearboxes often result in two types of fault modes during operation: pitting corrosion on tooth surfaces and cracks at tooth roots. Thus, how to accurately diagnose pitting and crack faults in planetary gearboxes is the main focus of this study.

### 2.2. Vibration Data Processing Based on Markov Image Coding

The Markov transition field (MTF) employs Markov transition probabilities to maintain the informational linkage across time domains. Firstly, the span value of the vibration signal is divided. The calculations are performed for the gaps between the separated value ranges. Then, following the temporal order of the vibration signal sampling points, the MTF matrix is computed based on the transition probability between the value range of the current data sampling location and that of the next sampling point, thereby preserving the time dependency and spectral characteristics of the vibration. Hence, the MTF is selected to transform the one-dimensional signal into a two-dimensional one.

The MTF represents a technique for encoding time series images, leveraging the Markov transition matrix. It views the evolution of a time series over time as a Markovian process. Under the condition that, given the known current state, its subsequent development is independent of its prior evolution. Thus, the Markov transfer matrix is constructed and then extended to the Markov transfer field to realize the image coding. Through this process, the vibration signal can be converted into a two-dimensional image. For the time series *X* = (*x_t_*, *t* = 1, 2, …, *T*), its image coding steps are as follows:

1. The time series *X*(*t*) is divided into Q equal parts according to time *t*, where the time series of the i equal part is denoted as *X_i_*(*t*) (*i* = 1, 2, …, *Q*), and the *N_i_* sampling points contained within the sequence of *X_i_*(*t*) are denoted as *x_i,j_* in chronological order (*j* = 1, 2, …, *N_i_*);
2. The range of the sampling point is denoted as [*X_L_*, *X_U_*], and is equally divided into *Q* intervals, and the *k*th interval is denoted as *q_k_* = [*X_kL_*, *X_kU_*], *X_L_* = *X_1L_*,*X_U_* = *X_QU_*, where *k* (*k* = 1, 2, …, *Q*);
3. The quantity of sampling points, *x_i,j_*, in *X_i_*(*t*) falling into the interval *q_k_* = [*X_kL_*, *X_kU_*] is denoted as *Z_ik_*;
4. Calculate *w_ik_* = *Z_ik_*/(*N*_1_ + *N*_2_ + … *N_Q_*), traverse all *i* and *k*, and construct the transition matrix.

(1) $$W=\left[\begin{array}{ccc} \omega_{11} & \ldots & \omega_{1Q}\\ \omega_{21} & \cdots & \omega_{2Q}\\ \vdots & \ddots & \vdots \\ \omega_{Q1} & \cdots & \omega_{QQ} \end{array}\right]\;s.t.\;\sum_{j}\omega_{ik}=1$$

The MTF can be used to convert one-dimensional sequences into time-dependent two-dimensional feature maps, which can preserve the time-dependent and frequency features of the original one-dimensional signals. Different transition probabilities lead to variations in MTF image pixels, which can be used as input to better leverage the advantages of residual networks for image classification.

The resolution of the image generated by the MTF is *Q* × *Q*, in which each pixel can be represented by *x_ik_*, the calculation method of *x_ik_* is *ω_ik_* × 255, and thus a matrix, ***x***, is formed, and expressed as follows:

(2) $$x=\left[\begin{array}{ccc} x_{11} & \ldots & x_{1Q}\\ x_{21} & \cdots & x_{2Q}\\ \vdots & \ddots & \vdots \\ x_{Q1} & \cdots & x_{QQ} \end{array}\right]\;s.t.\;x_{ik}\in \{0,255\}$$

In this experiment, each fault includes mild, moderate, and severe levels. Each group of experiments is divided into three speed conditions: 20 Hz, 30 Hz, and 40 Hz. Under each speed condition, ten sets of data are measured and each set of data can be transformed into a pixel matrix using the MTF method, wherein the pixel matrix is recorded as *x*_1_, *x*_2_, …, *x_a_*, …, *x_n_* (*a* ≤ *n*).

## 3. Intelligent Diagnostic Model for Planetary Gearboxes

**Figure 1:**
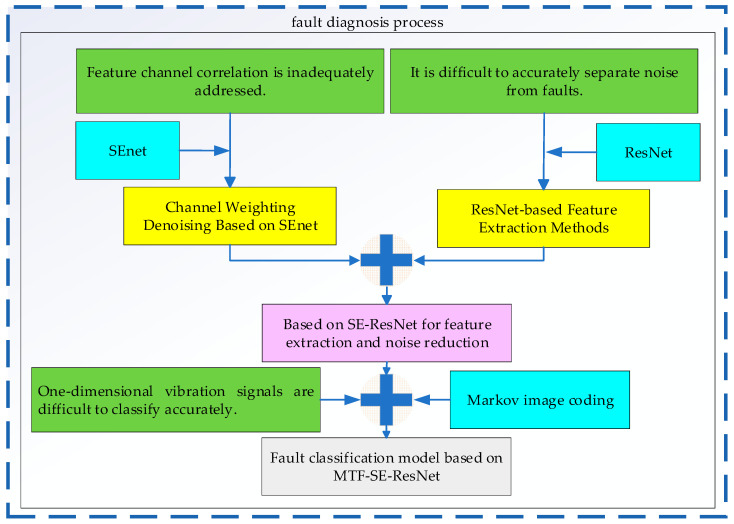
Diagram of fault diagnosis process.

This section proposes a fault diagnosis method based on MTF-SE-ResNet. Firstly, the SE-ResNet network is formed by embedding SEnet into ResNet to address the issues of insufficient channel correlation and separating fault features from noise. Subsequently, the data processed by MTF (Multi-scale Time-Frequency analysis, assumed context based on the acronym) is input into the SE-ResNet to constitute the MTF-SE-ResNet classification model, which solves the problem of accurately identifying fault types from one-dimensional signals input under a strong noise background. See Figure 1.

### 3.1. ResNet-Based Feature Extraction Methods

The vibration signals of planetary gearboxes contain a significant amount of noise. These noise characteristics can be learned by the network with the increasing layers, but the accuracy decreases. This paper utilizes a residual network instead of a convolutional neural network to extract fault features.

**Figure 2:**
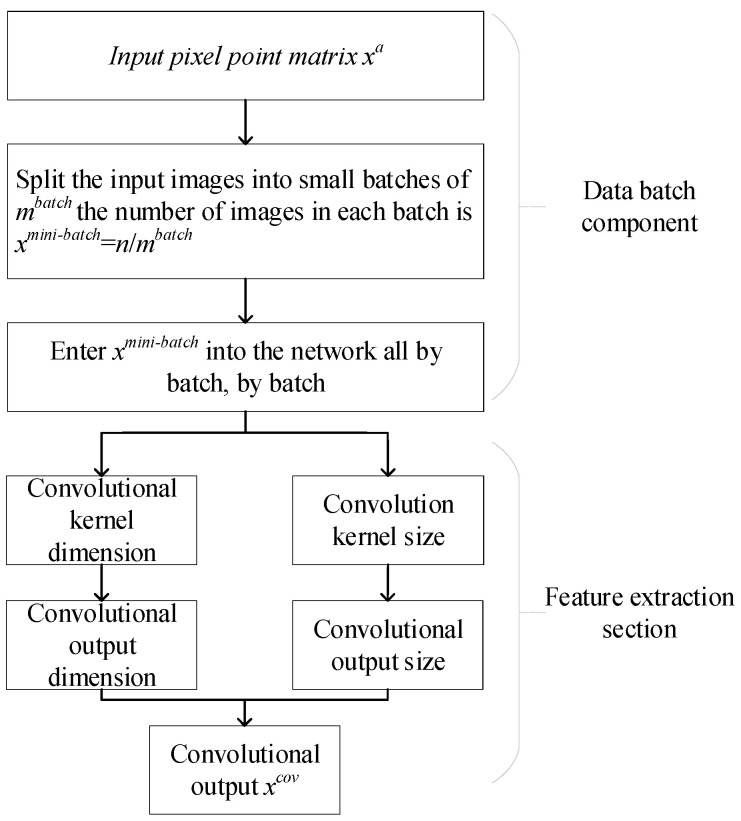
Convolution process.

The residual neural network consists of a direct mapping part and a residual part. The residual learning module is primarily composed of convolutional layers, batch normalization (BN) layers, and Rectified Linear Unit (ReLU) layers. The convolutional layer performs convolution operations on local regions of the input image using convolution kernels with different sizes, and traverses the image with a certain stride to form multiple different fault feature maps. Given an input *x^a^*, where *a* ∈ *Z* [1, *n*], the process of extracting features through the convolutional part is illustrated in Figure 2.

The mathematical formula of the convolution process is shown in Equation (3):

(3) $$x^{a\mathrm{cov}}=\left(\sum x^{a}\ddot{*}k\right)$$

where *x^acov^* denotes the result of the computation completed by the matrix convolution of the *a*th input pixel point, then *x^cov^* consists of *x^acov^*, and *x^a^* denotes the input pixel point matrix; *k* denotes the convolution kernel that connects the input feature maps to the output feature maps, and the convolution kernel length and width (*H* and *W*) and dimension (*C*) are set artificially, but the elements of the convolution kernel matrix are generated by a computer; and * denotes the matrix convolution operation.

The dimension of the convolution output *x^acov^* is determined by Equation (4). The input image is *Q* × *Q*, the convolution kernel size is *F* × *F*, the step stride is *S*, and the padding is *P,* which is the number of pixels filled, and then the size of the output feature map is shown below:

(4) $$x^{acov}=\left[\left(Q-F+2P\right)\;/S\right]+1$$

The BN layer primarily compresses the features extracted by the convolutional layers, retaining only the salient features relevant to fault diagnosis and discarding irrelevant ones. This is crucial for enhancing classification accuracy. Within the neural network, BN transforms the input data into a normal distribution with a mean of 0 and a variance of 1, thereby accelerating convergence speed and improving model precision. By incorporating learnable parameters *γ* and *β*, the BN layer restores the feature distribution that the original network aims to learn. The forward propagation formula for the BN layer is presented in Equation (5):

(5) $$\left\{\begin{array}{c} \mu =\frac{1}{m_{\mathrm{batch}}}\sum_{m_{\mathrm{batch}}}{x_{r}}^{\mathrm{cov}}\\ \sigma^{2}=\frac{1}{m_{\mathrm{batch}}}\sum_{m_{\mathrm{batch}}}{\left({x_{r}}^{\mathrm{cov}}-\mu \right)}^{2}\\ x^{n}=\frac{{x_{r}}^{\mathrm{cov}}-\mu }{\sqrt{\sigma^{2}+\varepsilon }}\\ y^{n}=\gamma x^{n}+\beta \end{array}\right.$$

where *m_batch_* refers to the size of the small batch, *x^n^ and y^n^* are the input and output of the *N*th observation in the small batch, *ε* is a constant close to 0 to ensure numerical stability, *γ* is the scaling parameter, and *β* is the bias parameter.

Following batch normalization, activation is necessary, and popular choices for activation functions encompass Sigmoid, Tanh, and ReLU. The activation function serves to introduce nonlinear factors, mapping fault features that surpass a threshold value to the output through the function, thereby achieving the objective of feature extraction. The proposed model employs the modified linear unit activation function, ReLU, which is defined as:

(6) σ(x)=max(x,0)

The expression of the activation is:

(7) $${x_{r}}^{\mathrm{cov}}=\sigma ({x^{cov}}_{p})$$

The ResNet incorporates a convolutional component, enabling it to extract features. By preserving a portion of the original input information, ResNet alters the learning objective and mitigates the issue of classification accuracy saturation. By directly incorporating shallow features into the extraction of deep features, ResNet is proved to be more adept at extracting signal features compared with CNNs.

**Figure 3:**
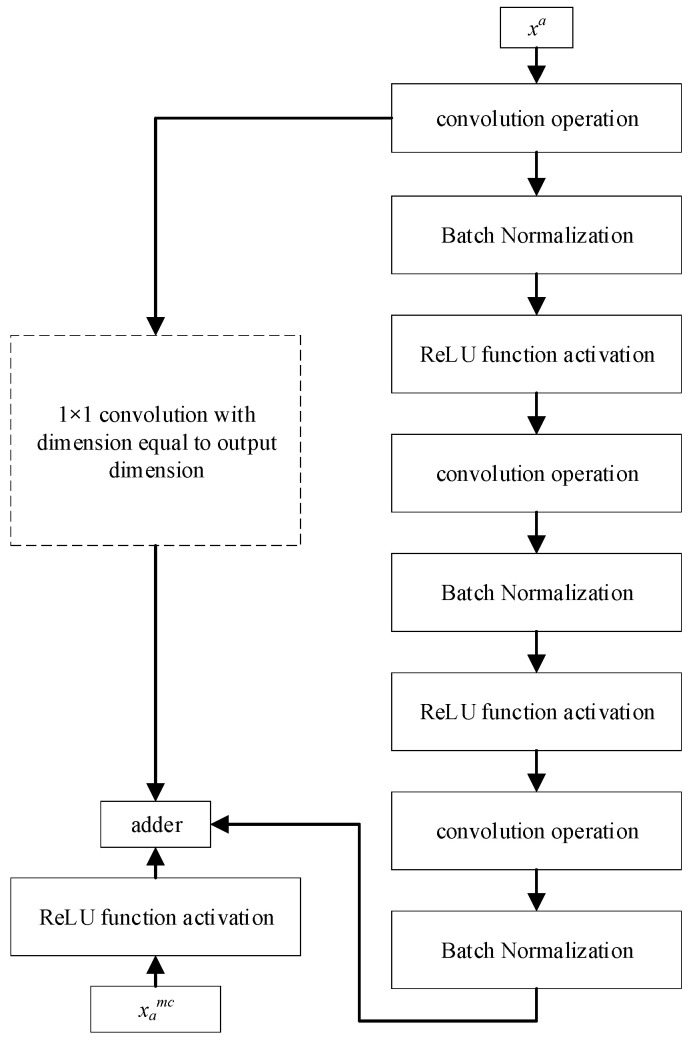
Residual block.

The residual network is composed of a series of residual blocks, as shown in Figure 3. The residual block is expressed as:

(8) $$x^{cn}=x^{a}*J_{1\times 1}+F(x^{a},W^{n})$$

The residual block is divided into two parts: the direct mapping part and the residual part. The direct mapping reacts to *x^a^* ∗ *J*_1×1_, where *J*_1×1_ represents a 1 × 1 convolution kernel, *F*(*x_l_*,*W_l_*) is the residual part, which generally consists of two or three convolution operations, and *x_a_^mc^* is the output of the residual block.

In cases where the dimensions of *x^a^* and *x_a_^mc^* differ, a 1 × 1 convolutional module is required and is incorporated into the left branch. The output from one residual block, denoted as *x_a_*^1*c*^, serves as the input for the subsequent residual block. The cumulative output from all residual blocks is represented by *x_a_^mc^*, where *m* represents the number of residual blocks.

**Figure 4:**
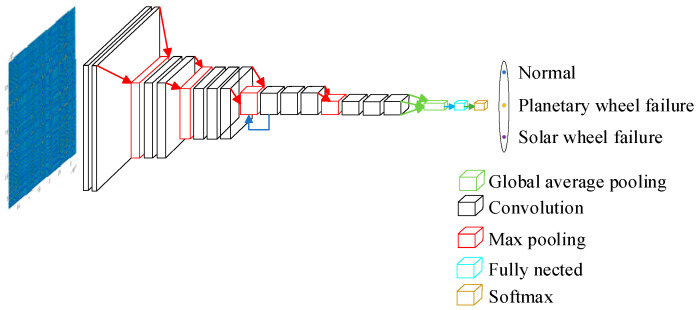
Residual network structure.

ResNet34 is adopted as the residual network framework for fault diagnosis. The network commences with a convolutional operation utilizing a 7 × 7 kernel and a stride of 2 × 2, followed by a max pooling layer. The central section is organized into four distinct layers: layer [1], layer [2], layer [3], and layer [4], with each layer containing 3, 4, 6, and 3 residual blocks, respectively. The number of convolutional filters progressively increases from 64 in the first layer to 128, 256, and finally 512 in the fourth layer. Each layer employs 3 × 3 convolutional kernels with a stride of 1 × 1. The overall network architecture is depicted in Figure 4.

In the last part, the global average pooling output classification probability is calculated as follows,

(9) $$y^{mc}=\frac{1}{H\times W}\sum_{i=1}^{H}\sum_{k=1}^{W}{x_{b}}^{mc}(i,k)$$

where *m* denotes the total count of feature maps outputted by the residual blocks, *y^mc^* represents the one-dimensional vector, and *x_b_^mc^* signifies the element located on the *b*th feature map (with 1 ≤ *b* ≤ 512) within the output of the final residual block. As the number of layers in traditional neural networks increases, learning the identity function becomes increasingly challenging, often leading to network degradation and suboptimal training outcomes. In the context of planetary gearbox fault diagnosis, the accuracy of traditional neural networks typically exhibits an initial rise followed by a decline as the network layers increase. This is because deeper layers tend to capture not only the relevant vibration signal features but also unwanted noise.

### 3.2. Channel Weighting Denoising Based on SEnet

By enabling adaptive learning on the channels within the residual blocks, the SE attention mechanism assigns weights to each channel. The noisy channels tend to have small weights, which further diminish the impact of strong noise signals on the network’s output, thereby enhancing the overall robustness and accuracy of the fault diagnosis.

The squeeze-and-excitation (SE) channel attention mechanism adjusts the activation values of each channel in the input feature map by adaptively learning the importance weights of each channel [21]. The SE module is primarily structured into two stages: squeeze and excitation. The SE module recognizes the varying degrees of importance among channel information and adaptively learns a specific value for each channel. This value is intimately tied to the classification features encapsulated within each channel. Given that noisy channels generally exhibit weaker features, the squeeze stage can be interpreted as a form of noise reduction, where the value assigned to *C* represents the significance of that particular channel. The squeeze stage involves compressing the spatial dimensions of the feature map through the application of global average pooling. This algorithm aggregates the spatial information across each channel, reducing the feature map to a single-dimensional vector that retains only the channel dimension *C*. This process effectively condenses the spatial scale, enabling the network to focus on the relationships between channels rather than the spatial details within each channel, and serves as a mechanism to highlight the importance of each channel for the subsequent excitation stage.

The SE module is inserted after the first residual block of the layer [3], and the calculation formula is shown as Equation (10):

(10) $$F_{s}=\frac{1}{H\times W}\sum_{i=1}^{H}\sum_{k=1}^{W}{x_{256}}^{8c}(i,k)$$

where *H* × *W* is the size of the feature map, *x_a_^mc^*(*i*,*k*) is the pixel value of position (*i*,*k*) in the channel of the feature graph with shape and size *H* × *C*, and *F_s_* is the response after the compression operation.

The excitation stage of the SE module initially reduces the input dimension *C* via a primary fully connected layer. This reduction not only enhances the model’s ability to discern crucial features and mitigate noise, but also minimizes the number of parameters within the FC layers, thereby improving the module’s efficiency [22]. Subsequently, dimension *C* is reinstated through the activation of the ReLU function, followed by a secondary fully connected layer. This reinstatement process further elaborates on the inter-channel relationships in a nonlinear fashion. Ultimately, the channel features transform the sigmoid function, yielding weights for each channel ranging from 0 to 1. These weights signify the relative significance of each channel. By multiplying these weights with the original channel features, the SE module generates new and weighted features that serve as the ultimate output. This methodology achieves differential treatment of channel features, emphasizing the most pertinent while de-emphasizing less relevant or noisy ones. The computational formulation of this process, as indicated in Equation (11), can be articulated as follows:

(11) $$F_{e}=sig(M_{2}\sigma (M_{1}F_{s}))$$

where sig is the Sigmoid function, *M*_1_ is the first fully connected layer, *M*_2_ is the second fully connected layer, *σ* is the activation function ReLU, and *F_e_* is the excitation response.

**Figure 5:**
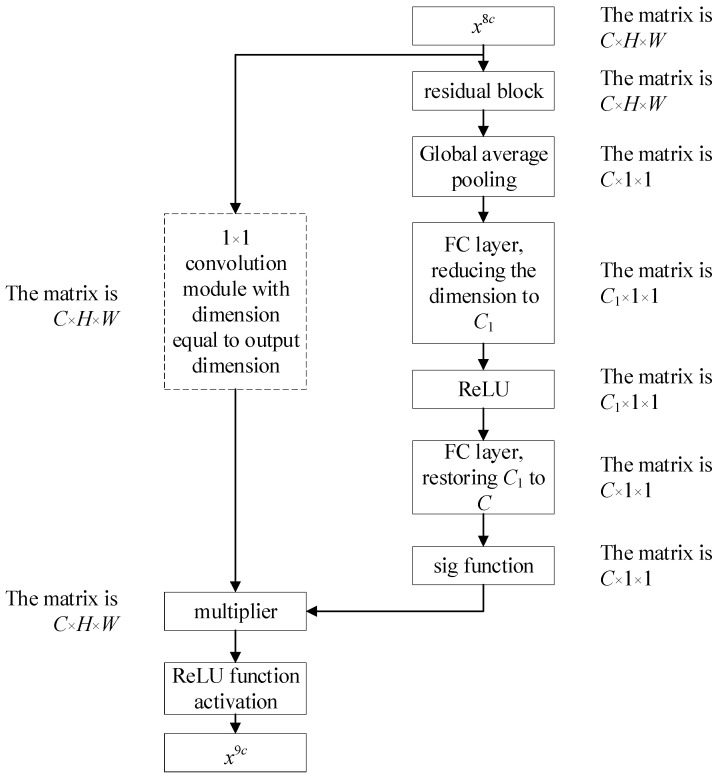
Residual attention module.

In essence, the residual attention module integrates the SE module into the residual module of the residual block to further extract features and eliminate the influence of noise on the network, ensuring high accuracy even under strong noise conditions. In this article, the SE module is inserted after the first residual block in the layer [3]. The residual attention module is illustrated as follows (Figure 5).

### 3.3. Fault Classification Model Based on MTF-SE-ResNet

**Figure 6:**
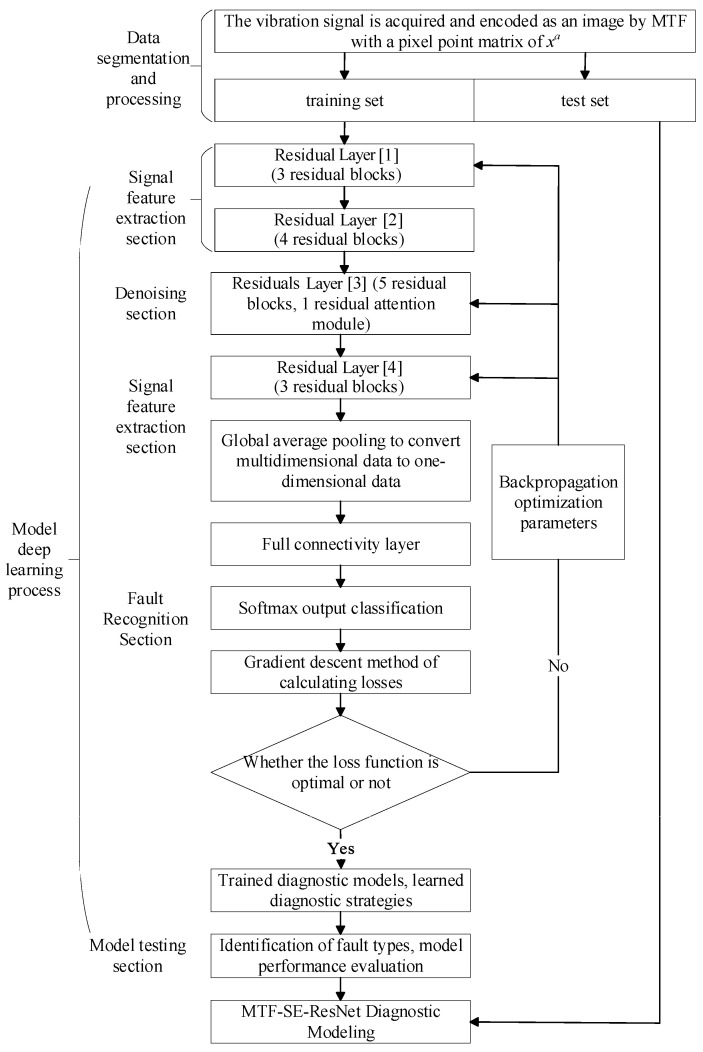
MTF-SE-ResNet model.

The fault diagnosis model of the planetary gearbox based on MTF coding and the SE-ResNet network can directly extract features from the original vibration signals. Firstly, the vibration signal MTF is encoded to generate a two-dimensional image. Then, the SE-ResNet model is built and trained by input images. The specific steps of the bearing fault diagnosis method based on MTF-SE-ResNet are as follows (Figure 6):

(1) Segmentation of planetary gearbox vibration signals: vibration signals are segmented according to a certain data length.
(2) Conversion and data enhancement: the segmented vibration signals are converted into 2D images using MTF, followed by data enhancement.
(3) Model construction: under the premise that the original Resnet34 network remains unchanged, the fault diagnosis model MTF-SE-ResNet is obtained by inserting the residual attention module after the 1st residual block in the layer [3].
(4) Input and feature extraction: the 2D images are divided into training and test sets and input into the MTF-SE-ResNet network to extract fault information.
(5) Fault diagnosis: fault diagnosis is achieved by global average pooling and mapping the results to fault types using the Softmax function.

**Figure 7:**
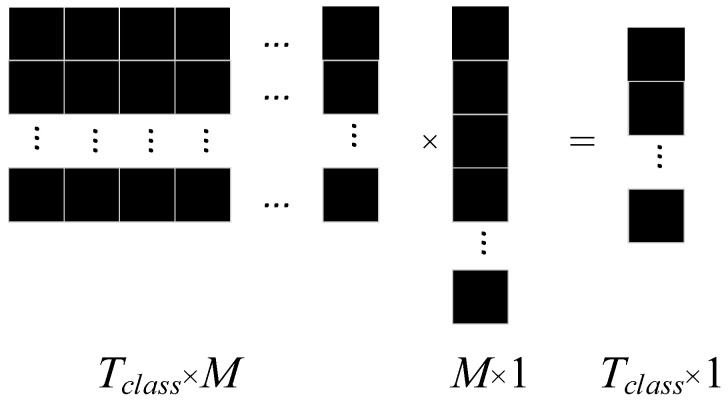
Schematic diagram of the full connectivity layer.

The function of the fully connected layer is to combine the output feature map vectors of global average pooling into a feature map matrix (*T_class_* × *M*), and then multiply an *M* × 1 vector to become the classification vector we need, where *M* is the number of global average pooling layers, and *T_class_* is the number of categories we will eventually divide. The expression is as follows (Figure 7).

The purpose of the Softmax function is to convert all the vector elements in the *T_class_* × 1 vector output by the fully connected layer into a value between (0, 1), which can represent the output probability of the class Softmax. The Softmax function is calculated as follows:

(12) $$S_{f}=\frac{e^{f}}{e^{g}}$$

where *f* denotes the element whose position in *T* × 1 is *f* and *e^g^* is the e-index of sum of the *T_class_* × 1 vector elements.

When doing classification training, it is necessary to label the category of classification. If a sample belongs to the second category, the output value of the output node corresponding to this category should be 1, while the output value of other nodes is 0, that is, [0, 0, 1, 0 … 0, 0], which is the most expected output of the neural network. We use the crossover loss function to measure the difference between the actual output of the network and the correct label, and use this difference to update the network parameters through backpropagation. The loss function selected in this paper is the cross entropy loss function, expressed as follows:

(13) $$H(p,q)=-\sum_{x}(p(x)logq(x))$$

where *p*(*x*) is the distribution of the input and *q*(*x*) is the distribution of the output.

## 4. Experimental Validation and Analysis

To verify the validity of the proposed diagnostic model, pytorch1.13.1 and cuda11.6 frameworks were used as programmed diagnostic models, run on Windows 12th Gen Intel(R) Core(TM) i5-12400F CPU and NVIDIA GeForce GTX 3060Ti GPU graphics card. The health status of the planetary gearbox was simulated by the SQ fault diagnosis comprehensive test bed, and vibration signals were collected for test verification and analysis.

### 4.1. Test Platforms

**Figure 8:**
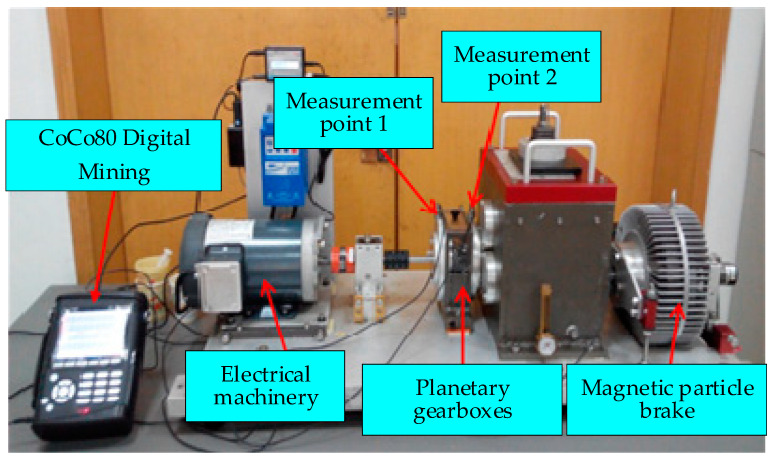
Composition of the test bench and arrangement of measurement points.

The fault simulation test for the planetary gearbox was conducted utilizing the SQ power transmission fault diagnosis comprehensive test rig, as depicted in Figure 8. This test rig encompasses a drive motor, a coupling, a two-stage planetary gearbox, a parallel shaft gearbox, and a magnetic brake. The purpose of the experiment was to simulate and analyze faults within the first stage of the planetary gearbox, specifically tooth surface pitting and root cracking of the planetary gear and sun gear. For data acquisition, CoCo80 digital sampling was employed to capture both the first-level and second-level vibration signals emanating from the planetary gearbox, along with the motor speed signals. The precise locations of the measurement points are indicated in Figure 8. The experimental setup was configured with a sampling frequency of 25.6 kHz, ensuring high-resolution data capture. The acceleration sensor sensitivity was set to 50 mv/g for enhanced sensitivity to subtle vibrations, while the magnetic powder brake was operated at a voltage of 14.0 V and a current of 1.23 A to provide a controlled load condition. The experiment incorporated two types of faults for the first stage of the planetary gearbox: tooth surface pitting and root cracking of the planetary gear and sun gear. Each fault type was further categorized into mild, moderate, and severe levels, allowing for a comprehensive analysis of fault progression. To assess the impact of operational speed on fault signatures, each experimental group was conducted under three distinct speed conditions: 20 Hz, 30 Hz, and 40 Hz. This approach ensured that the fault characteristics were thoroughly investigated across a range of operating speeds.

### 4.2. Data Description

**Figure 9:**
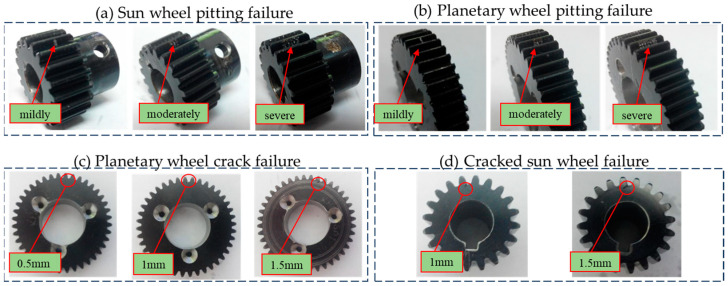
Planetary gearbox fault forms.

During the experiment, the vibration data of the planetary gearbox were meticulously collected under 12 distinct health conditions, each tested at three different rotating speed–load combinations: 20 Hz, 30 Hz, and 40 Hz. This approach allowed for a comprehensive analysis of the gearbox’s performance across varying operational conditions. For each health condition and speed setting, the vibration signals were segmented into 10 non-overlapping samples, each with a length of 32,768 data points. Consequently, under each working condition, a total of 327,680 samples were accumulated, providing a rich dataset for subsequent analysis. Figure 9 visually illustrates the various fault forms and their severity levels, offering a clear understanding of the conditions under which the vibration data were collected. These faults, including tooth surface pitting and root cracking of the planetary gear and sun gear at mild, moderate, and severe levels, were precisely simulated to mimic real-world scenarios. By analyzing the vibration signatures under these conditions, valuable insights into the behavior and degradation of the planetary gearbox were gained.

### 4.3. Data Preprocessing

**Figure 10:**
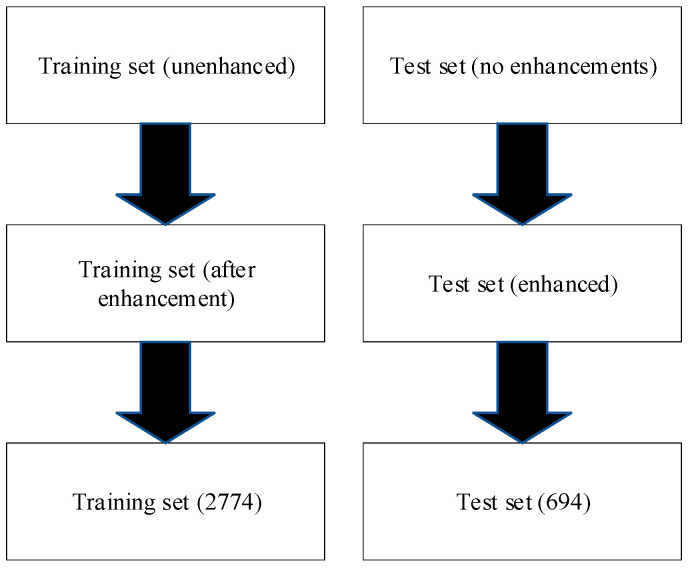
Data segmentation chart.

**Figure 11:**
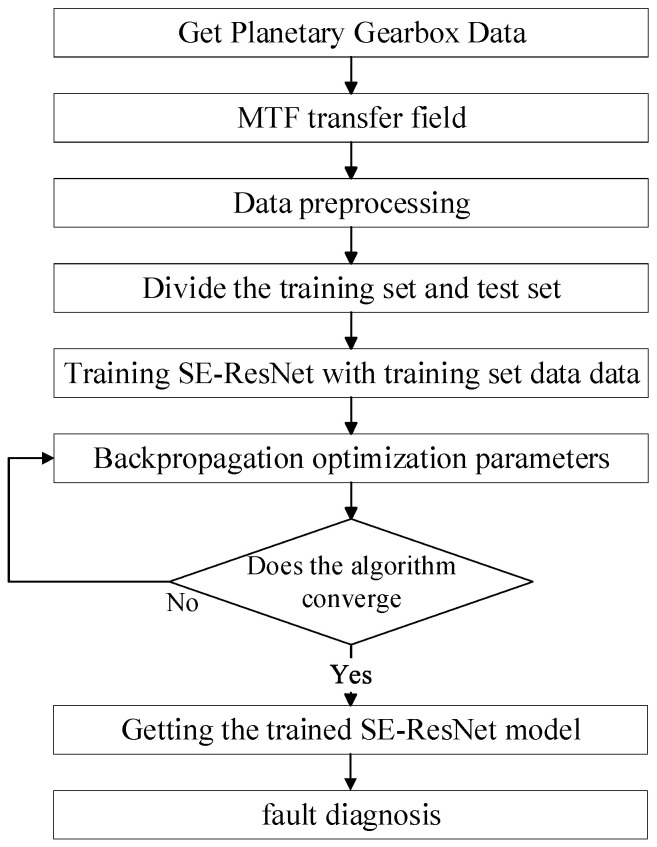
Logical block diagrams of the MTF-ResNet algorithm.

Given the limited number of samples obtained from the experiment, specifically 30 trouble-free images, 90 solar wheel failure images, and 80 planetary wheel failure images, it was necessary to employ data augmentation techniques to enhance the dataset for training and testing purposes. This was done to ensure that the model had sufficient data to learn from, particularly for the underrepresented classes, and to provide robust fault knowledge for the test set. In this paper, various data augmentation techniques were applied to increase the number of images in each class. For the trouble-free class, techniques such as flipping (both horizontally and vertically), brightening, darkening, and possibly other transformations were employed to increase the number of images from 30 to 992. Similarly, the number of solar wheel failure images was augmented from 90 to 919, and the number of planetary wheel failure images from 80 to 864. The test set was also augmented, resulting in 248 trouble-free images, 229 solar wheel failures, and 216 planetary wheel failures. The enhanced dataset was then divided into training and test sets according to an 8:2 ratio, with the training set used to train the proposed MTF-SE-ResNet model and the test set used to evaluate its performance. Figure 10 and Figure 11 illustrate the specific steps involved in the data preprocessing and final fault diagnosis process. Figure 10 likely depicts the data preprocessing stage, where the raw images are subjected to various augmentation techniques to create an expanded dataset. This expanded dataset is then divided into training and test sets, with the training set further utilized to train the MTF-SE-ResNet model. Figure 11, on the other hand, shows the fault diagnosis process, where the trained MTF-SE-ResNet model is applied to the test set to classify the images into their respective fault categories. The model’s performance is evaluated based on its ability to accurately identify the fault type in each image.

### 4.4. Comparison and Analysis of Results

**Figure 12:**
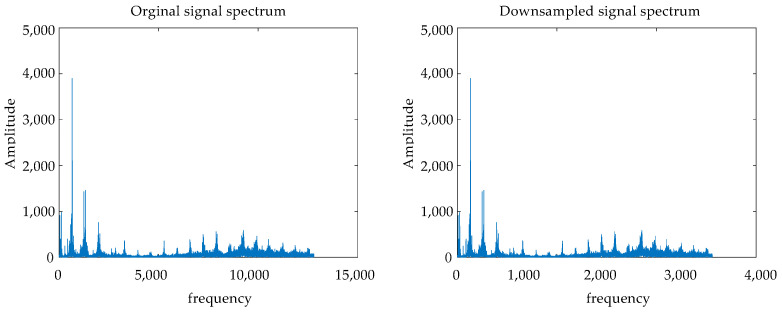
Comparison of original and downsampled signal spectra.

First, the normal signal is downsampled at the sampling frequency of fs = 6400 Hz. The spectrum of the downsampled signal in Figure 12 right and the original spectrum in Figure 12 left are shown. It can be seen from the figure that the high-frequency noise of the original signal is removed after downsampling, which proves that there is a large amount of noise.

**Figure 13:**
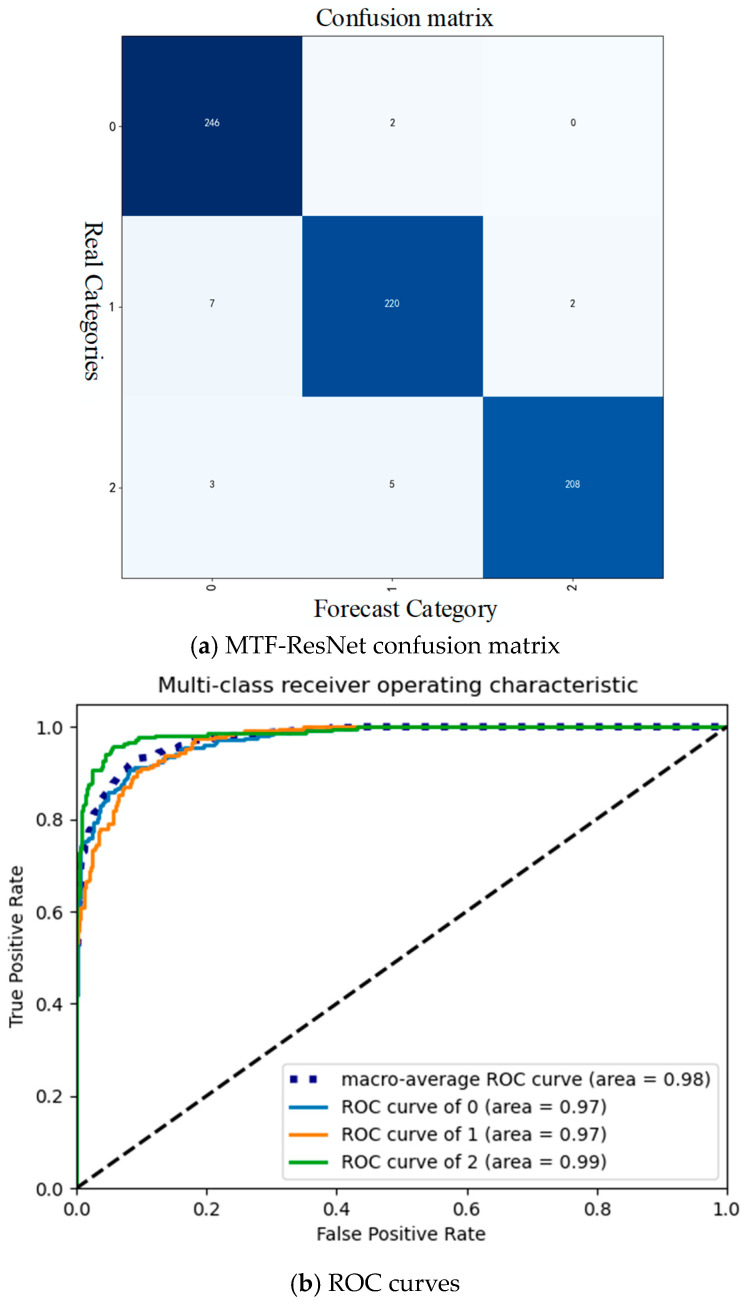
MTF-ResNet confusion matrix with ROC curves.

The results, presenting the confusion matrix in Figure 13a and ROC curve in Figure 13b, provide strong evidence that the proposed MTF-SE-ResNet model has a high classification accuracy and reliability for diagnosing faults in a planetary gearbox. The horizontal axis represents the predicted label categories, while the vertical axis represents the true label categories. Label 0 indicates normal signals, label 1 indicates pitting faults, and label 2 indicates crack faults. The confusion matrix, which summarizes the performance of the model by comparing the predicted labels with the true labels, shows only a few misclassifications. This indicates that the model can accurately distinguish the three classes of signals: normal signals, solar wheel fault signals, and planetary wheel fault signals. The low number of misclassifications is a good sign that the model has learned the distinguishing features of each class effectively. The ROC curve further reinforces the high accuracy of the model by providing a graphical representation of the trade-off between the true positive rate and the false positive rate at various threshold settings. The fact that the area under the ROC curve for the normal signal and planetary wheel fault signal is 97%, and 99% for the solar wheel fault, indicates that the model can achieve a high level of classification accuracy with a low false alarm rate. The average accuracy of 98% across all classes is also a testament to the robustness and effectiveness of the proposed method. Overall, the results presented in Figure 13 demonstrate that the MTF-SE-ResNet model is a reliable and accurate tool for diagnosing faults in planetary gearboxes. The high classification accuracy and low misclassification rate make it a promising approach for real-world applications where early detection and accurate diagnosis of gearbox faults are crucial for maintaining equipment health and preventing costly downtime.

**Figure 14:**
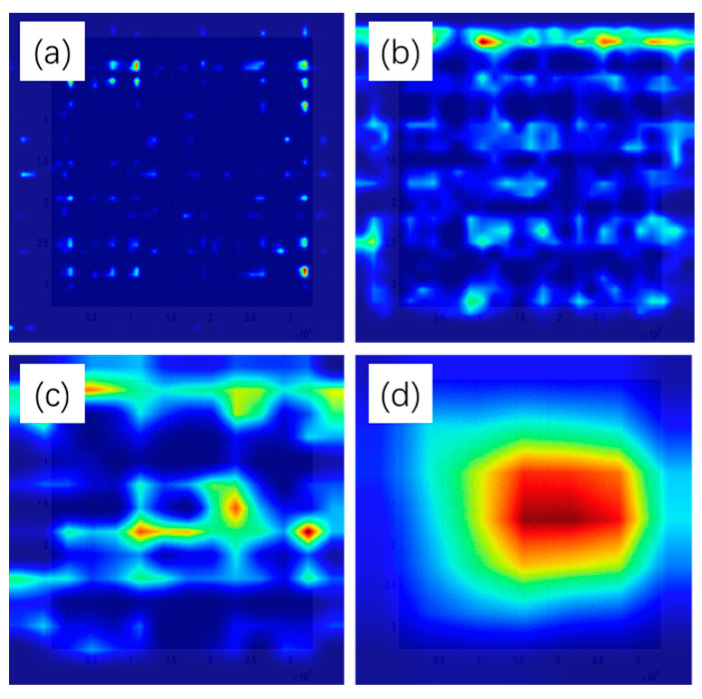
Heat map of the nodes of the residual network of solar wheel failures. (**a**) represents the output heatmap of the first Residual Block, (**b**) represents the output heatmap of the second Residual Block, (**c**) represents the output heatmap of the third Residual Block, and (**d**) represents the output heatmap of the fourth Residual Block.

By examining the thermal maps of the network’s output at each residual layer, we can obtain a deep understanding of how the model learns and extracts features from the input MTF images of solar wheel faults in the planetary gearbox. The red part represents the data portion that exhibits fault characteristics in the planetary gearbox. The larger the range of the red appearance is, the more thorough the fault extraction is at that residual layer, leading to more accurate output results. The thermal maps at the first residual layer show relatively scattered image features, indicating that the model is initially extracting surface-level features such as textures and details; this is shown in Figure 14a. This is consistent with the nature of ResNet, which tends to learn simple, low-level features in the early layers and then builds upon those to extract more complex, high-level features in the deeper layers. As the network layer increases, the image features become more abstract and representative, as evidenced by the thermal maps at the subsequent residual layers; this is shown in Figure 14b,c. This suggests that the model can capture more meaningful and discriminative features that are useful for distinguishing between different fault types. The observation that only a few features are activated (red) in the fully connected layer, while most remain inactive (dark blue), is also noteworthy; this is shown in Figure 14d. This indicates that the model has learned to select and focus on the most relevant features for the classification task, while ignoring irrelevant or redundant information. This ability to automatically select and weight features is a key strength of deep neural networks and contributes to their high performance in many complex tasks.

Overall, the visualization of the SE-ResNet network’s output at different layers provides valuable insights into the feature extraction process of the MTF-SE-ResNet model. It confirms that the model can learn and extract meaningful features from the input MTF images, and that these features become more abstract and representative as the network goes deeper. This understanding can help improve the model’s performance further by optimizing the network architecture or training process.

**Figure 15:**
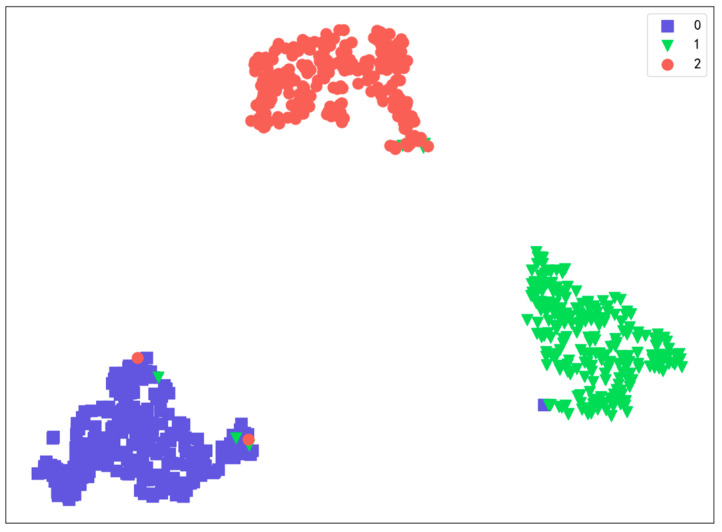
T-SNE visualization of feature dimensionality reduction.

To further explore the process of MTF-SE-ResNet feature extraction, the data were input into MTF-SE-ResNet, and the final extracted features were visualized through T-distributed Stochastic Neighbor Embedding (T-SNE) for dimensionality reduction. Figure 15 shows the classification of the original data after they passed through ResNet34 network. As can be seen from the figure, the classification characteristics of the original data gradually become clear after they pass through the network structure of each layer, and they show obvious classification characteristics in the fully connected layer.

To further validate the accuracy of the model, the proposed algorithm in this paper was employed to test the experimental data, and was compared with three other classification methods: CNN, MTF-CNN, and ResNet34. Both CNN and MTF-CNN utilized the classic convolutional neural network AlexNet [23]. All three methods were trained using transfer learning, where the training set consisted of 2774 randomly selected images from both the source domain and target domain samples, and the test set comprised 694 randomly selected images from the target domain. The proposed MTF-SE-ResNet method was compared against these three methods. The accuracy rates of the four methods on the test set are illustrated in Figure 15, with orange stripes representing CNN accuracy, green for MTF-CNN, purple for ResNet34, and yellow for the method proposed in this paper.

**Figure 16:**
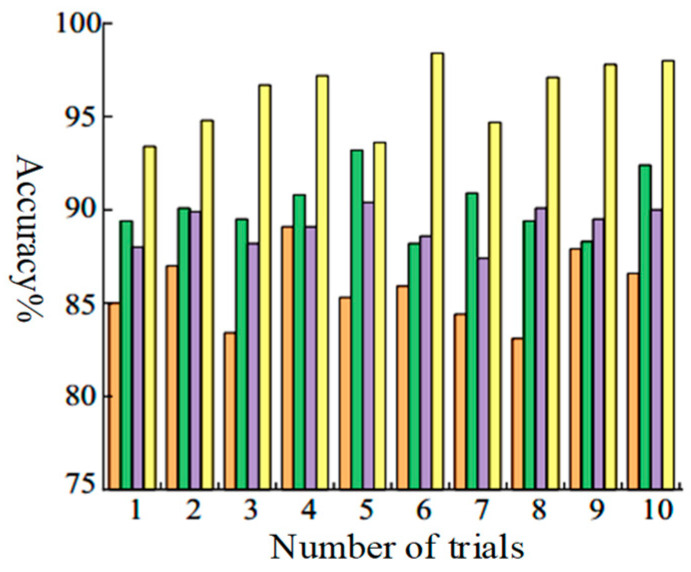
Fault classification accuracy analysis results for 10 tests on planetary gearboxes.orange stripes representing CNN accuracy, green for MTF-CNN, purple for ResNet34, and yellow for the method proposed in this paper.

As can be seen from Figure 16, the highest accuracy of the proposed method is 98.1%, the lowest is 93%, and the average is 96.5%. In comparison, the CNN attained the highest accuracy level of 87.1%, the lowest level of 83%, and an average level of 85.3%. For the MTF-CNN model, the highest accuracy is 93.5%, the lowest is 88.1%, and the average is 89.6%. In addition, the highest accuracy of ResNet34 is 91.6%, the lowest is 87.2%, and the average is 88.3%. The ResNet34 network can reduce the influences of noise on accuracy, but its poor performance in feature extraction from one-dimensional signals results in a lower accuracy compared with MTF-CNN. Based on the results of ten sets of experiments, the developed MTF-SE-ResNet diagnostic model demonstrates excellent performance in planetary gearbox fault diagnosis; it effectively addresses the issues resulting from the heavy noise and varying vibration signal distributions during the planetary gearbox operation under complex working conditions.

## 5. Conclusions

The proposed MTF-SE-ResNet model, combined with transfer learning, transforms one-dimensional signals into two-dimensional images, and enables the residual network to effectively extract abstract features from the vibration signal images. By incorporating the SE attention mechanism, the proposed method effectively mitigates the impact of noise on diagnostic results, and exhibits outstanding performance in gearbox fault diagnosis. The main conclusions are as follows:

(1) Fault diagnosis of the planetary gearbox can be transformed into an image recognition task by utilizing the MTF to convert one-dimensional vibration signals into two-dimensional vibration images. As a result, the accuracy and stability of fault diagnosis can be significantly improved using ResNet.
(2) The issue that deep network layers inadvertently learn noise as features is effectively resolved by inserting the SE attention mechanism into the traditional residual network.
(3) Comparative experiments have demonstrated that the proposed method achieves a maximum classification accuracy of 98.1% and an average classification accuracy of 96.5%. This proves that the proposed method meet the engineering application requirements of planetary gearboxes under strong noise backgrounds.

## Data Availability

The data presented in this study are available on request from the corresponding author. Due to privacy concerns, we choose not to provide our data publicly.

## References

[1] Wang S., Nejad A., Bachynski E.E., Moan T. (2021). A comparative study on the dynamic behaviour of 10 MW conventional and compact gearboxes for offshore wind turbines. Wind Energy.

[2] Kim J.-G., Park Y.-J., Lee G.-H., Lee S.-D., Oh J.-Y. (2018). Experimental study on the carrier pinhole position error affecting dynamic load sharing of planetary gearboxes. Int. J. Precis. Eng. Manuf..

[3] Gao M., Shang Z., Li W., Liu F., Pang H., Liu J. (2023). Analysis of Wear Mechanism and Fault Characteristics of Planet Gears with Multiple Wear Types in Planetary Gearbox. J. Vib. Eng. Technol..

[4] Ding C., Zhao M., Lin J. (2020). Sparse feature extraction based on periodical convolutional sparse representation for fault detection of rotating machinery. Meas. Sci. Technol..

[5] Song Y., Liu Z., Gao S. (2024). Current Collection Quality of High-speed Rail Pantograph-catenary Considering Geometry Deviation at 400 km/h and Above. IEEE Trans. Veh. Technol..

[6] D’Elia G., Mucchi E., Cocconcelli M. (2017). On the identification of the angular position of gears for the diagnostics of planetary gearboxes. Mech. Syst. Signal Process..

[7] Wang X., Si S., Li Y. (2020). Multiscale diversity entropy: A novel dynamical measure for fault diagnosis of rotating machinery. IEEE Trans. Ind. Inform..

[8] Li Y., Wang S., Yang Y., Deng Z. (2022). Multiscale symbolic fuzzy entropy: An entropy denoising method for weak feature extraction of rotating machinery. Mech. Syst. Signal Process..

[9] Milovančević M., Nikolić V., Petkovic D., Vracar L., Veg E., Tomic N., Jović S. (2018). Vibration analyzing in horizontal pumping aggregate by soft computing. Measurement.

[10] Pang X., Xue X., Jiang W., Lu K. (2020). An investigation into fault diagnosis of planetary gearboxes using a bispectrum convolutional neural network. IEEE/ASME Trans. Mechatron..

[11] Li Y., Du X., Wan F., Wang X., Yu H. (2020). Rotating machinery fault diagnosis based on convolutional neural network and infrared thermal imaging. Chin. J. Aeronaut..

[12] Wang D.-F., Guo Y., Wu X., Na J., Litak G. (2020). Planetary-gearbox fault classification by convolutional neural network and recurrence plot. Appl. Sci..

[13] Scholtyssek J., Bislich L.J., Cordes F., Krieger K.-L. (2023). Vibration-Based Detection of Bearing Damages in a Planetary Gearbox Using Convolutional Neural Networks. Appl. Sci..

[14] Chen R., Huang X., Yang L., Xu X., Zhang X., Zhang Y. (2019). Intelligent fault diagnosis method of planetary gearboxes based on convolution neural network and discrete wavelet transform. Comput. Ind..

[15] Xu Y., Yan X., Sun B., Zhai J., Liu Z. (2021). Multireceptive field denoising residual convolutional networks for fault diagnosis. IEEE Trans. Ind. Electron..

[16] Wang Z., Wang J., Zhao Z., Wang R. (2017). A novel method for multi-fault feature extraction of a gearbox under strong background noise. Entropy.

[17] Zhao M., Kang M., Tang B., Pecht M. (2017). Deep residual networks with dynamically weighted wavelet coefficients for fault diagnosis of planetary gearboxes. IEEE Trans. Ind. Electron..

[18] Zhang K., Tang B., Deng L., Tan Q., Yu H. (2021). A fault diagnosis method for wind turbines gearbox based on adaptive loss weighted meta-ResNet under noisy labels. Mech. Syst. Signal Process..

[19] Xin P., Yang K.X., Wen X.Q. (2023). Research on wind turbine fault diagnosis method based on improved SE-CNN. J. Jilin Inst. Chem. Technol..

[20] Wu Y., Yang F., Liu Y., Zha X., Yuan S. A comparison of 1-D and 2-D deep convolutional neural networks in ECG classification. Proceedings of the IEEE Engineering in Medicine and Biology Society.

[21] Xu M.Q., Wang Y.Q. (2021). An imbalanced fault diagnosis method for rolling bearing based on semi- supervised conditional generative adversarial network with spectral normalization. IEEE Access.

[22] Hu J., Shen L., Sun G. Squeeze-and-excitation networks. Proceedings of the IEEE Conference on Computer Vision and Pattern Recognition.

[23] Krizhevsky A., Sutskever I., Hinton G.E. (2017). ImageNet classification with deep convolutional neural networks. Commun. ACM.

